# Alkaline Phosphatase PhoD Mutation Induces Fatty Acid and Long-Chain Polyunsaturated Fatty Acid (LC-PUFA)-Bound Phospholipid Production in the Model Diatom *Phaeodactylum tricornutum*

**DOI:** 10.3390/md21110560

**Published:** 2023-10-26

**Authors:** Kaidian Zhang, Jiashun Li, Jie Cheng, Senjie Lin

**Affiliations:** 1 State Key Laboratory of Marine Resource Utilization in the South China Sea, School of Marine Biology and Fisheries, Hainan University, Haikou 570228, China; 2 State Key Laboratory of Marine Environmental Science, College of Ocean and Earth Sciences, Xiamen University, Xiamen 361102, China; 3 School of Life Sciences, Liaocheng University, Liaocheng 252000, China; chengjie@lcu.edu.cn; 4 Department of Marine Sciences, University of Connecticut, Groton, CT 06340, USA

**Keywords:** alkaline phosphatase, microalgae, fatty acids, CRISPR/Cas9 genome editing, lipid composition, PhoD

## Abstract

With rapid growth and high lipid contents, microalgae have become promising environmentally friendly candidates for renewable biodiesel and health supplements in our era of global warming and energy depletion. Various pathways have been explored to enhance algal lipid production, especially gene editing. Previously, we found that the functional loss of PhoD-type alkaline phosphatase (AP), a phosphorus-stress indicator in phytoplankton, could lead to increased lipid contents in the model diatom *Phaeodactylum tricornutum*, but how the AP mutation may change lipid composition remains unexplored. This study addresses the gap in the research and investigates the effects of PhoD-type AP mutation on the lipid composition and metabolic regulation in *P. tricornutum* using transcriptomic and lipidomic analyses. We observed significantly modified lipid composition and elevated production of fatty acids, lysophosphatidylcholine, lysophosphatidylethanolamine, ceramide, phosphatidylinositol bisphosphate, and monogalactosylmonoacylglycerol after *PhoD_45757* mutation. Meanwhile, genes involved in fatty acid biosynthesis were upregulated in mutant cells. Moreover, the mutant exhibited increased contents of ω-3 long-chain polyunsaturated fatty acid (LC-PUFA)-bound phospholipids, indicating that *PhoD_45757* mutation could improve the potential bioavailability of PUFAs. Our findings indicate that AP mutation could influence cellular lipid synthesis and probably redirect carbon toward lipid production and further demonstrate that AP mutation is a promising approach for the development of high-value microalgal strains for biomedical and other applications.

## 1. Introduction

In face of the rising demand for energy and food, microalgae with fast growth, high protein, and high lipid contents have received intensifying interest in both bioenergy and health products [1,2]. Algae are carbon-neutral and renewable resources for both energy and high-value products [3]. Microalgae grow rapidly (doubling within one day), and their oil content can reach up to 25–75% of their dry weight [4]. The biodiesel production per unit area from microalgae is conservatively estimated to be more than 10 times that of typical terrestrial oil crops [5]. Therefore, oleaginous algae may now be the most promising environmentally friendly candidates for renewable biodiesel [3,4]. Moreover, algal lipids are surprisingly diverse and unique in structure, including valuable species such as triglycerides (TAGs) and long-chain polyunsaturated fatty acids (LC-PUFAs) [6], which can serve as a source of biofuel and health supplements, respectively.

LC-PUFAs, including ω-3 and ω-6 LC-PUFAs, are indispensable nutrients that cannot be synthesized de novo by the human body [7]. Omega-3 LC-PUFAs such as eicosapentaenoic acid (EPA) and docosahexaenoic acid (DHA) have been shown to have health benefits on cardiovascular, lactation, brain, and visual functions and have thus been used as a nutraceutical additive [8,9]. Meanwhile, it has been shown that DHA-bound lysophosphatidylcholine (LPC) is the preferred carrier form of DHA in the brain [10]. As for ω-6 LC-PUFAs, arachidonic acid (ARA) is an important component of biological membranes and a precursor of various bioactive lipids and eicosanoids (e.g., prostaglandins) [11,12]. Thus, ARA and its metabolites have important roles in skeletal muscle, the nervous system, and the immune system against allergies and parasites [12]. In addition, the application of algal lipids in cosmetics [13] and other industrial fields is emerging [14]. Given the great application prospects of algal lipids, ways to enhance lipid production have been a hot topic of research.

Multiple pathways have been reported to enhance microalgal lipid production. Firstly, the growth conditions, especially nutrient conditions, could be manipulated to modulate the cellular lipid profile of phytoplankton [15,16,17]. Lipid abundance and composition in algae can be changed by nitrogen stress, phosphorus starvation, and high-iron conditions [18,19,20]. For instance, phosphorus nutrient (P) deficiency could reprogram membrane lipid composition (replacing phospholipids with sulfolipids and nitrolipids) and significantly increase the lipid contents of algae [21,22,23]. Secondly, increased lipid production can also be achieved by altering the expression of genes involved in the lipid biosynthesis pathway through molecular techniques. The suppression or overexpression of glycerol-3-phosphate acyltransferase (GPAT), long-chain acyl-CoA synthetase (LACS), acyl-CoA:diacylglycerol acyltransferase (DGAT), and 1-acyl-sn-glycerol-3-phosphate acyltransferase (AGPAT1) could elevate algal lipid biosynthesis [24,25,26,27]. Thirdly, with the aid of gene silencing (e.g., RNA interference) and knockout/knockdown techniques (e.g., CRISPR-Cas9), researchers have found that the functional loss of certain genes not directly involved in lipid metabolic pathways can also affect oil yield [17]. For instance, significant increases in cellular lipid synthesis were observed after gene silencing of UDP-glucose pyrophosphorylase, nitrate reductase, and phosphoenolpyruvate carboxykinase in the model diatom *Phaeodactylum tricornutum* [17,28,29]. Recently, we found that the mutation of two different types of the alkaline phosphatase (AP) gene (both PhoA and PhoD), a P-stress indicator, could increase the lipid content in *P. tricornutum* [30,31]. However, it is not clear which lipid components were increased after AP null mutation.

Thanks to recent advances in analysis tools such as mass spectrometry, lipidomics has grown rapidly [32,33]. Particularly, the combination of lipidomics with transcriptomics analysis provides a powerful tool to deepen our understanding of phytoplankton lipid metabolism [25]. In this study, using combined transcriptomics-lipidomic analysis, we investigated the effects of PhoD-type AP (*PhoD_45757*) mutation on lipid composition and metabolic regulation in *P. tricornutum*. We observed significantly promoted lipid production and modified lipid composition after *PhoD_45757* mutation, especially the induced production of LC-PUFAs and ω-3 (LC-PUFA)-bound phospholipids in *P. tricornutum*. Correspondingly, we also observed the upregulation of genes involved in fatty acid biosynthesis. With these findings, we demonstrate the high potential of AP mutagenesis for the development of high-value microalgae for health supplements and other applications.

## 2. Results

### 2.1. Lipidomic Profile in mPhoD Cells

**Figure 1:**
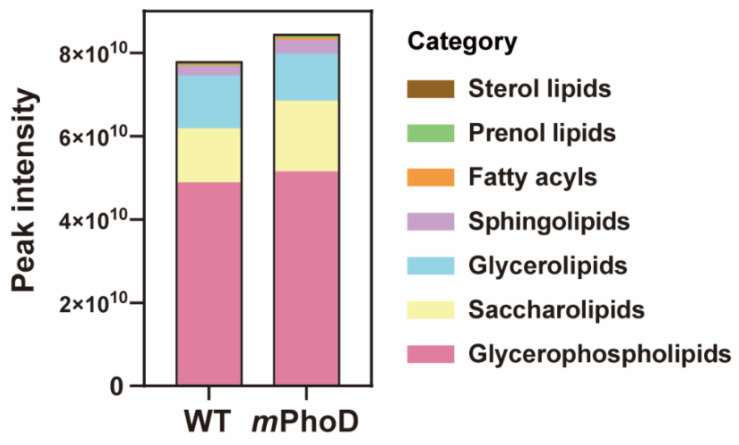
The content and components of the different lipid categories in WT and *m*PhoD cells. The lipid categories were divided based on LIPID MAPS. The average peak intensity values of each lipid molecule detected from each sample were calculated at the level of category and subclass and log2 converted.

Generally, the lipidomic profile dramatically changed after *PhoD_45757* mutation (Figure 1 and Table S1). First, the total lipid content showed an 8% increase in *PhoD_45757* mutant (*m*PhoD) cells compared to wild-type (WT) cells (*t*-test, *p* < 0.01) (Figure 1). In total, 439 lipid molecules were identified in *P. tricornutum* (Table S1) and further classified into seven categories based on the lipid metabolites and pathways strategy (LIPID MAPS) lipid classification system, including fatty acyls (6), glycerolipids (53), gylcerophospholipids (171), prenol lipids (1), saccharolipids (188), sphingolipids (16), and sterol lipids (4) (Figure 1 and Table S1). Among the seven categories, fatty acyls, sphingolipids, saccharolipids, prenol lipids, and glycerophospholipids showed 2.0-fold, 0.4-fold, 0.3-fold, 0.2-fold, and 0.05-fold higher contents in *m*PhoD cells, respectively (*t*-test, *p* < 0.01 in saccharolipids, sphingolipids, and fatty acyls) (Figure 1).

**Figure 2:**
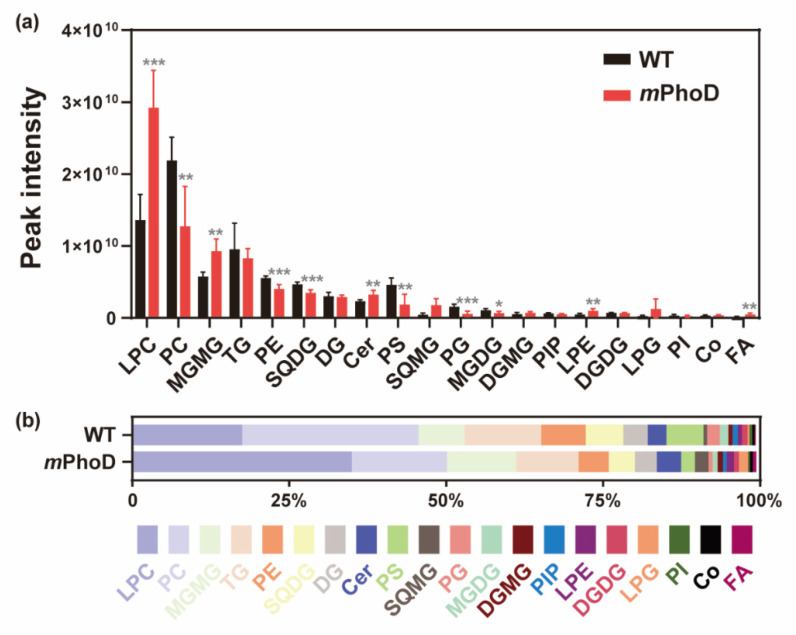
The contents (**a**) and proportion (**b**) of different lipid subclasses in the WT and *m*PhoD cells. Only the lipid subclasses with the top 20 contents are shown. LPC, lysophosphatidylcholine; PC, phosphatidylcholine; MGMG, monogalactosyl monoacylglycerol; TG, triglyceride; PE, phosphatidylethanolamine; SQDG, sulfoquinovosyl diacylglycerol; DG, diglyceride; Cer, ceramide; PS, phosphatidylserine; SQMG, sulfoquinovosyl monoacylglycerol; PG, phosphatidylglycerol; MGDG, monogalactosyl diacylglycerol; DGMG, digalactosyl monoacylglycerol; PIP, phosphatidylinositol phosphate; LPE, lysophosphatidylethanolamine; DGDG, digalactosyl diacylglycerol; LPG, lysophosphatidylglycerol; PI, phosphatidylinositol; Co, coenzyme; FA, fatty acid. * *p* < 0.05; ** *p* < 0.01; *** *p* < 0.001.

At the level of lipid subclasses, *PhoD_45757* mutation significantly induced the production of lysophosphatidylcholine (LPC), monogalactosylmonoacylglycerol (MGMG), ceramide (Cer), lysophosphatidylethanolamine (LPE), fatty acids (FAs), phosphatidylinositol bisphosphate (PIP2), and lysophosphatidylinositol (LPI) (*t*-test, *p* < 0.05 in all comparisons) (Figure 2 and Table S1). The most abundant lipid subclass in *m*PhoD cells was LPC, with a 1.1-fold increase compared to that observed in WT cells (*t*-test, *p* < 0.001) (Figure 2a). Meanwhile, LPC accounted for up to 34.88% of the total lipid content in *m*PhoD cells, which was also significantly higher than that in the WT cells (17.45%) (*t*-test, *p* < 0.001) (Figure 2b). Moreover, the contents of MGMG, Cer, LPE, FA, PIP2, and LPI in the *m*PhoD cells were 0.6-fold, 0.4-fold, 1.0-fold, 2.2-fold, 11.7-fold, and 1.1-fold more enhanced than those in the WT cells, respectively (*t*-test, *p* < 0.05) (Figure 2a). Accordingly, the proportion of MGMG, Cer, and FA in *m*PhoD cells was significantly higher than that in the WT cells (*t*-test, *p* < 0.05) (Figure 2b).

### 2.2. Differential Lipids and Lipid Metabolism-Related DEGs in the mPhoD/WT Comparison

**Figure 3:**
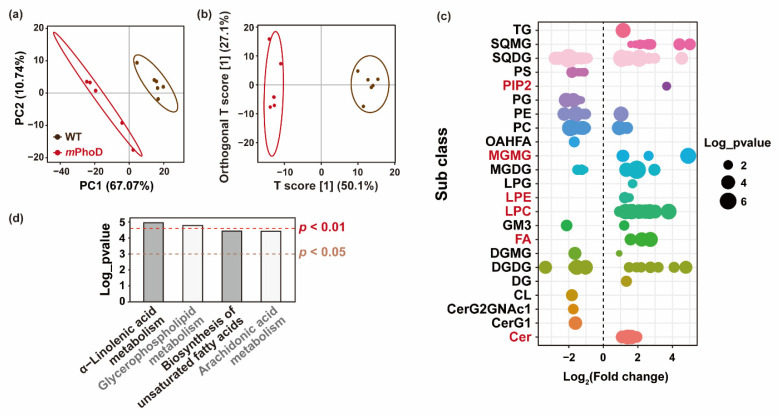
Differential lipids in the *m*PhoD/WT comparison. PLS-DA (**a**) and OPLS-DA (**b**) results of the WT and *m*PhoD groups. Ellipses represent 95% confidence intervals. (**c**) General distribution of 174 differential lipids in the *m*PhoD/WT comparison. Each point represents one differential lipid. Red fonts indicate significantly increased lipid subclasses in the *m*PhoD cells. (**d**) Significantly enriched KEGG pathway of differential lipids detected in the *m*PhoD/WT comparison. Abbreviations: TG, triglyceride; SQMG, sulfoquinovosyl monoacylglycerol; SQDG, sulfoquinovosyl diacylglycerol; PS, phosphatidylserine; PIP2, phosphatidylinositol bisphosphate; PG, phosphatidylglycerol; PE, phosphatidylethanolamine; PC, phosphatidylcholine; OAHFA, (O-acyl)-1-hydroxy fatty acid; MGMG, monogalactosyl monoacylglycerol; MGDG, monogalactosyl diacylglycerol; LPG, lysophosphatidylglycerol; LPE, lysophosphatidylethanolamine; LPC, lysophosphatidylcholine; GM3, ganglioside; FA, fatty acid; DGMG, digalactosyl monoacylglycerol; DGDG, digalactosyl diacylglycerol; DG, diglyceride; CL, cardiolipin; CerG2GNAc1, simple Glc series; CerG1, monogylcosylceramide; Cer, ceramide.

Using partial least-squares discriminant analysis (PLS-DA) based on the detected lipids in *P. tricornutum*, the principal components 1 and 2 together can explain 77.81% of the overall variance (Figure 3a). Furthermore, orthogonal partial least-squares discriminant analysis (OPLS-DA) was used to establish the relationship between lipid contents and algal strains (WT and *m*PhoD) and calculate the variable important for the projection (VIP) to identify differentially changed lipids in the *m*PhoD cells (Figure 3b). Both PLS-DA and OPLS-DA showed large differences in lipidome between *m*PhoD and WT (Figure 3a,b). In total, 174 lipid species were significantly differentially regulated in the *m*PhoD/WT comparison, including 88 induced and 86 depressed lipid species (Figure 3c and Table S2). Among them, all 37 lipid species belonging to PIP2 (1), MGMG (6), LPE (2), LPC (20), FA (3), and Cer (5) were all induced in the *m*PhoD/WT comparison (Figure 3c). Moreover, these 37 differential lipids showed a positive correlation with each other (Figure S1). The differential lipids were significantly enriched to four KEGG pathways, including α-linolenic acid metabolism, glycerophospholipid metabolism, the biosynthesis of unsaturated fatty acids, and arachidonic acid metabolism (*p* < 0.05) (Figure 3d).

**Figure 4:**
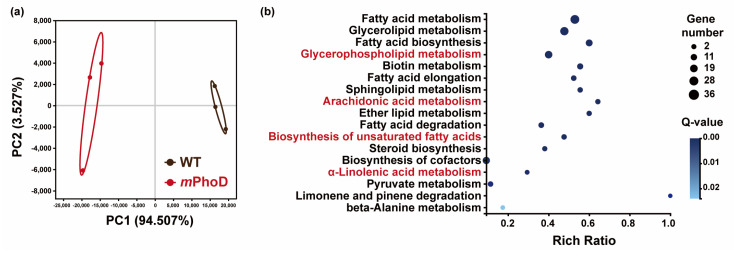
Transcriptomic responses after *PhoD_45757* mutation. (**a**) PCA results of the WT and *m*PhoD groups. Ellipses represent 95% confidence intervals. (**b**) Significantly enriched KEGG pathway of DEG involved in lipid metabolism in the *m*PhoD/WT comparison. Red fonts indicate four pathways significantly enriched by the differential lipids.

Besides the lipidome, the transcriptomic analysis of WT and *m*PhoD cells was also conducted to identify transcriptomic regulations on lipid metabolism after *PhoD_45757* mutation. A great difference in transcript levels after *PhoD_45757* mutation was clearly reflected in the PCA analysis (Figure 4a). A total of 118 DEGs were detected as participating in lipid metabolism in *P. tricornutum* (Table S3). These DEGs were significantly enriched to 17 lipid metabolic pathways including fatty acid metabolism, glycerolipid metabolism, sphingolipid metabolism, and four pathways significantly enriched by the differential lipids (Figure 4b).

### 2.3. Increased FA, PUFAs, LPC, PIP2, and Cer Synthesis in Both Transcriptomic and Lipidomic Levels after PhoD_45757 Mutation

**Figure 5:**
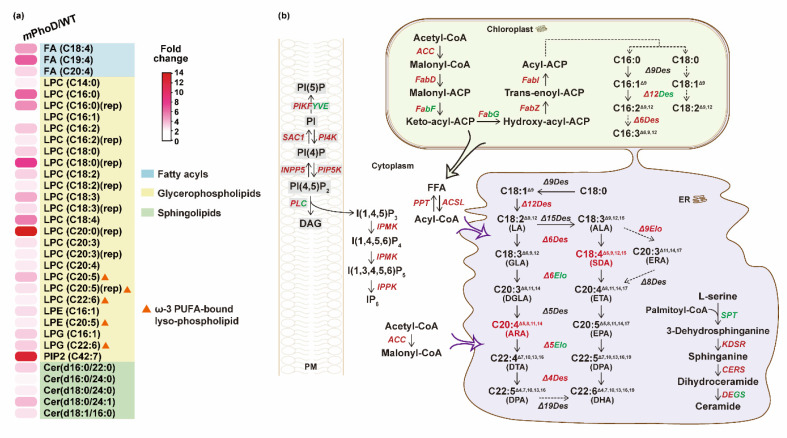
Upregulated fatty acid synthesis and other lipid metabolic regulation in *m*PhoD cells. (**a**) Increase in differential lipid species belonging to FA, LPC, LPE, LPG, PIP2, and Cer in *m*PhoD cells. (**b**) Upregulated fatty acid synthesis, phosphatidylinositol signaling system, and ceramide synthesis in *m*PhoD cells. Red and green fonts indicate upregulation and downregulation in *m*PhoD cells, respectively. PM, plasma membrane; ER, endoplasmic reticulum. The detailed information on genes shown in this figure can be found in Table S4.

According to the increased FA content, we observed significant increases in three polyunsaturated fatty acid (PUFA) species in the *m*PhoD/WT comparison, including arachidonic acid (ARA, C20:4) which displayed 3.0-fold upregulation and stearidonic acid (SDA, C18:4) which displayed with 4.7-fold upregulation (Figure 5a). Meanwhile, key enzymes throughout the fatty acid biosynthesis process showed upregulation in *m*PhoD cells, including acetyl-CoA carboxylase (ACC), [acyl-carrier-protein] S-malonyltransferase (FabD), 3-hydroxyacyl-[acyl-carrier-protein] dehydratase (FabZ), and enoyl-[acyl-carrier protein] reductase (FabI) (Figure 5b). Moreover, the acyl-lipid Δ12 desaturase (Δ12Des), Δ6 desaturase (Δ6Des), Δ4 desaturase (Δ4Des), and very-long-chain enoyl-CoA reductase (Δ9Elo) performing PUFA synthesis in the chloroplast and endoplasmic reticulum (ER) showed upregulation in *m*PhoD cells (Figure 5b).

As the most abundant subclass in *m*PhoD cells, all 20 LPC species were significantly increased after *PhoD_45757* mutation (Figure 5a). Notably, four ω-3 PUFA-bound LPCs were detected in the *m*PhoD/WT comparison, including one eicosatetraenoic acid (ETA)-LPC (LPC C20:4) with a 2.4-fold increase, two eicosapentaenoic acid (EPA)-LPCs (LPC C20:5) with 2.7- and 3.8-fold increases, and one docosahexaenoic acid (DHA)-LPC (LPC C22:6) with 2.3-fold increase in the *m*PhoD cells (Figure 5a). Moreover, two more ω-3 PUFA-bound lysophospholipids, EPA-LPE (LPE C20:5) and DHA-LPG (LPG C22:6), were significantly induced after *PhoD_45757* mutation (Figure 5a).

As crucial signaling molecules and cell membrane components, PIP2 and Cer were induced after *PhoD_45757* mutation (Figure 5a). PIP2 showed up to a 12.7-fold increase in *m*PhoD cells, while five differential Cer species were upregulated by 2.1 to 3.8 folds in the *m*PhoD/WT comparison (Figure 5a). Meanwhile, we also noticed the strong transcriptomic regulation of the phosphatidylinositol signaling system after *PhoD_45757* mutation (Figure 5b). The interaction between membrane phosphatidylinositol phosphates (PIs) was activated by the upregulation of PI4K (phosphatidylinositol 4-kinase), phosphatidylinositol 4-phosphatase (SAC1), 1-phosphatidylinositol-4-phosphate 5-kinase (PIP5K), and inositol polyphosphate 5-phosphatase (INPP5B) in the *m*PhoD cells (Figure 5b). Meanwhile, the synthesis of IP_6_ from IP_3_ was promoted in the *m*PhoD cells as shown by the upregulation of inositol-polyphosphate multikinase (IPMK) and inositol-pentakisphosphate 2-kinase (IPPK) (Figure 5b). Moreover, ceramide synthesis was promoted as 3-dehydrosphinganine reductase (KDSR) and very-long-chain ceramide synthase (CERS) were upregulated in the *m*PhoD/WT comparison (Figure 5b).

## 3. Discussion

Microalgae are a promising source of fatty acids and lipids for human use as a health supplement [1]. Nutritional and environmental stresses can significantly affect cellular lipid composition [15]. Meanwhile, advances in genetic editing techniques and multi-omics studies have also greatly facilitated algal lipid research [34,35]. AP is a typical indicator of P-starvation and DOP utilization in phytoplankton. However, recently, we found that *PhoD_45757* (a PhoD-type AP) could potentially also regulate cellular nutrition homeostasis, cell cycle, and even lipid metabolism in *P. tricornutum* [30]. Meanwhile, researchers have proposed that altering the expression of genes not directly belonging to the lipid biosynthesis pathway can achieve carbon redirection toward lipid production [17]. In this study, increased lipid content and remodeled lipid composition in PhoD-mutated *P. tricornutum* were observed, among which the increases in LC-PUFAs and PUFA-bound lysophospholipids and ceramide were the most remarkable. Therefore, our results further confirm that *PhoD_45757* mutation could influence cellular lipid synthesis and probably redirect carbon toward lipid production. Because AP mutagenesis has become routine, this provides a promising approach for algal genetic engineering to improve lipid- and fatty acid-related traits in this and other genetically tractable algae.

### 3.1. Improved Ceramide Synthesis after PhoD_45757 Mutation

In the *m*PhoD cells, the ceramide content increased by 40%, and 5 of the 14 detected ceramide species were significantly increased (Figure 2 and Figure 5a, and Table S1). Moreover, the major genes (KDSR and CERS) involved in the biosynthesis of ceramide were upregulated after *PhoD_45757* mutation (Figure 5b). In the previous report, the ceramide content was <0.5% of the total lipids in the northern diatoms *Coscinodiscus concinnus* and *Porosira glacialis*, while it was 1.6% in *Chaetoceros socialis* [36]. Here, ceramide accounted for 3.9% of the total lipids in the *m*PhoD cells (3.0% in WT cells), which also showed a significant increase in the cellular proportion of ceramide after *PhoD_45757* mutation (Figure 2). Ceramide is an important member of the sphingolipids and plays an important role as a second messenger in cell signaling, cell differentiation, growth, and apoptosis [37,38]. We previously found that *m*PhoD cells showed a depressed cell cycle and decreased growth rate [30]. It has been reported that the accumulation of ceramide by the inhibitors of ceramide metabolism could lead to cell cycle arrest at G2–M [39]. Therefore, the enrichment of ceramide after *PhoD_45757* mutation indicates that *PhoD_45757* could probably modulate the cell cycle by regulating the cellular ceramide contents in *P. tricornutum* [30]. Furthermore, ceramides are now widely applied in cosmetic products for their skin moisturizing and barrier repair effects [14]. Thus, the enhancement of ceramide production can also benefit the application of the *PhoD_45757* mutant strain in cosmetic development. It is worth mentioning that, recently, ceramides isolated from red algae *Hypnea musciformis* were found to have anticancer effects and antiangiogenic and apoptotic effects in an experimentally induced mammary tumor [40]. Therefore, the potential of *PhoD_45757* mutagenesis in anticancer drug development is worth further exploring.

### 3.2. PhoD_45757 Mutation Could Improve the Production and Potential Bioavailability of PUFAs

*PhoD_45757* mutation promoted the expression of genes involved in fatty acid synthesis and led to a 2.2-fold increase in fatty acid content in *P. tricornutum* (Figure 2 and Figure 5). Accordingly, we observed upregulation of PUFA synthesis-related genes and increased PUFA contents (especially SDA and ARA) in *m*PhoD cells (Figure 5). ARA is one of the major components of brain membrane phospholipids and has great nutritional importance for muscle growth and brain health in the human body [12]. Therefore, the dramatic increase in ARA production here raises the possibility of exploiting the *PhoD_45757* mutant for ARA industrial production or dietary supplementation.

Moreover, lysophospholipids, especially LPC, significantly increased in the *m*PhoD cells (Figure 2). Among them, we noticed the abundant ω-3 LC-PUFA-enriched lysophospholipids (including LPC-EPA, LPC-DHA, LPE-EPA, and LPG-DHA) after *PhoD_45757* mutation (Figure 5a). Marine animals and plankton are the primary sources of ω-3 PUFA, and the ω-3 LC-PUFAs (in particular EPA and DHA) are of vital importance for human health [41,42]. The level of ω-3 LC-PUFA in the human body (mainly the brain) can be influenced by age, gender, and living habits, and researchers have found that dietary supplementation is effective in replenishing ω-3 LC-PUFA [43]. Moreover, compared to the ω-3 LC-PUFA in triacylglycerol forms, ω-3 LC-PUFA bound to phospholipid showed higher bioavailability and bioactivity [44]. As a DHA carrier, LPC-DHA can cross the blood–brain barrier and efficiently transport DHA into the brain, enriching brain DHA levels and improving memory [45,46]. Similarly, the dietary addition of LPC-DHA significantly increased retinal DHA in mice [47]. Thus, *PhoD_45757* mutant strains with enriched DHA/EPA-bound lysophospholipids can be considered as a potential resource for dietary supplementation of the brain EPA and DHA. This implies the potential to exploit *PhoD_45757* mutants for the dietary enhancement of brain function and the prevention of retinal disease. In addition, LPC-DHA has been recently reported to possess a cytotoxic effect on breast cancer cells [48]. Therefore, the induction of LPC-DHA by *AP* mutation is promising for biomedical applications.

## 4. Materials and Methods

### 4.1. Mutant Culture and Cell Collection

The *P. tricornutum* strain CCAP 1055/1 WT was obtained from the Culture Collection of Algae and Protozoa (Scottish Marine Institute, UK). The mutants of one PhoD-type AP (Phatr3_J45757, referred to as *PhoD_45757*) were created using CRISPR/Cas9 technology in our laboratory [49], and the strain with higher lipid fluorescence, *m*PhoD7, was selected for this experiment (hereafter *m*PhoD). Both WT and *m*PhoD were cultured in f/2 medium prepared with autoclaved 0.22 µm filtered seawater (salinity 30) at 20 °C with a photon flux of 100 µE m^−2^ s^−1^ under a 14:10 h light:dark cycle [30,50]. The batch culture experiment was set up with six biological replicates. After eight-day cultivation, >4 × 10^8^ cells from each culture were collected by centrifugation (5000 rpm, room temperature, 10 min). The obtained cell pellets were washed with PBS twice and stored in 50% glycerin at −80 °C.

### 4.2. Lipid Extraction and UPLC-MS Analysis

Firstly, mill magnetic beads, 800 µL of pre-chilled dichloromethane/methanol (3:1, *v*/*v*) precipitant, and 10 µL of the prepared internal standard were added to each sample. Then, the cell samples were ground using a TissueLyser for 5 min, sonicated in an ice bath for 10 min and left overnight in a −20 °C refrigerator. Next, 600 µL of supernatant was collected after centrifugation (25,000× *g*, 4 °C, 15 min) and lyophilized in a freeze drier. Subsequently, 120 µL of lipid-extracting solution (isopropyl alcohol: acetonitrile: water = 2:1:1) was added and fully mixed (10 min), and the mixture was centrifuged (25,000× *g*, 4 °C, 15 min).

A Waters UPLC I-Class Plus (Waters, Milford, MA, USA) Tandem Q Exactive High Resolution Mass Spectrometer (Thermo Fisher Scientific, Waltham, MA, USA) was used for lipid separation and detection (LC-MS/MS). Chromatographic separation was performed on a CSH C18 column (1.7 μm 2.1 × 100 mm, Waters, Milford, MA, USA). The positive ion mode consisted of mobile phase A (containing 60% acetonitrile in water, 10 mM ammonium formate, and 0.1% formic acid) and mobile phase B (consisting of 90% isopropanol, 10% acetonitrile, 10 mM ammonium formate, and 0.1% formic acid). The negative ion mode consisted of mobile phase A (containing 60% acetonitrile in water, 10 mM ammonium formate) and mobile phase B (containing 90% isopropanol, 10% acetonitrile, 10 mM ammonium formate). Elution was performed using the following gradients: 40~43% B over 0 to 2 min, 43~50% B over 2 to 2.1 min, 50~54% B over 2.1 to 7 min, 54~70% B over 7 to 7.1 min, 70~99% B over 7.1 to 13 min, 99~40% B over 13 to 13.1 min, held constant at 99~40% B over 13.1 to 15 min, and washed with 40% B over 13.1 to 15 min. The flow rate was 0.4 mL per min and the injection volume was 5 μL. The column temperature was maintained at 55 °C. Meanwhile, the primary and secondary mass spectrometry data acquisition was performed using Q Exactive (Thermo Fisher Scientific, Waltham, MA, USA). The full scan range was 70–1050 m z^−1^ with a maximum ion injection time of 100 ms and resolution of 70,000, and the automatic gain control (AGC) target for MS acquisitions was 3 × 10^6^. The top three precursors were selected for subsequent MSMS fragmentation with a maximum ion injection time of 50 ms and resolution of 17,500, and the AGC was 1e5. The stepped normalized collision energy was 15, 30, and 45 eV. The sheath gas flow rate and aux gas flow rate of the ESI were 40 and 10, respectively. The spray voltage (|KV|) of positive- and negative-ion modes were 3.80 and 3.20, respectively. The capillary temperature and aux gas heater temperature were 320 °C and 350 °C, respectively.

### 4.3. Lipid Identification and Annotation

The mass spectrometry data were analyzed using lipidsearch v.4.1 (Thermo Fisher Scientific, Waltham, MA, USA) software to identify and quantify the lipid molecules detected in the *m*PhoD and WT cells. Lipid classification was based on The Lipid Metabolites and Pathways Strategy (LIPID MAPS) Lipid Classification System. Then, the KEGG pathway database was used for the taxonomic and functional annotation of the identified lipids [51]. Furthermore, the overall differences in lipid species between *m*PhoD and WT were determined by partial least-squares–discriminant analysis (PLS-DA) and orthogonal partial least-squares discriminant analysis (OPLSDA) instead of traditional PCA. The fold change (FC) analysis and *p*-value were calculated by the *t*-test. Lipid species that met the following criteria can be considered as differential lipids: 1) variable importance in the projection (VIP) of the OPLS-DA model ≥ 1; 2) FC ≥ 1.2 or ≤0.83; and 3) *p*-value < 0.05. After that, the expression patterns and biological functions of the differential lipids in the *m*PhoD/WT comparison were further explored by pathway enrichment and correlation analyses.

### 4.4. Gene Expression Analysis and Differentially Expressed Genes Identification

Libraries for RNAseq were obtained from BioProject PRJNA841772 of the Short Read Archive (SRA) database in our previous report [30]. The latest genome annotation of *P. tricornutum* strain CCAP 1055/1 (ftp.ensemblgenomes.org, accessed on 17 September 2023) was used to perform the clean reads annotation using HISAT2 (v2.1.0) and Bowtie2 (v2.2.5) [52,53,54,55]. The gene expression level was quantified using RSEM (v1.2.8) [56]. Principal component analysis (PCA) was analyzed on the Majorbio Cloud platform [57]. Then, differentially expressed gene detection was based on Poisson distribution. False discovery rate (FDR) was used for the correction of the *p*-value to the *q*-value [58,59]. After that, genes with |log_2_ fold change (FC)| ≥1 and q-value ≤ 0.001 were defined as significantly differentially expressed genes (DEGs) in the *m*PhoD/WT comparison [60,61]. Using the phyper function in R software, the KEGG pathway enrichment analysis of DEGs with *q*-value ≤ 0.05 was regarded as significant enrichment.

### 4.5. Statistical Analysis

In this study, the samples for the lipidomics analysis were in sextuplicate (*n* = 6) and samples for the transcriptomics analysis were in triplicate (*n* = 3). Means and standard deviations were calculated based on the biological replicates. One-way analysis of variance (ANOVA) was carried out using SPSS 16.0 (IBM, Armonk, NY, USA) to assess the statistically significant differences between WT and *m*PhoD at the level of *p* < 0.05.

## 5. Conclusions

In this study, *PhoD_45757* mutation led to enhanced lipid production in *P. tricornutum*, making the mutant strain a promising candidate for health supplements. Among the lipid components, the increases in fatty acids, LC-PUFAs (especially ARA), PUFA-bound lysophospholipids, and ceramide were observed in mutant cells. The increased production of PUFA-bound lysophospholipids, especially PUFA-bound LPC, indicates the improvement of PUFA bioavailability after *PhoD_45757* mutation. Overall, the potential of AP mutation in microalgae lipids for biofuel and biomedical applications is well established, and its widespread application may be on the horizon.

## Supplementary Materials

The following supporting information can be downloaded at: https://www.mdpi.com/article/10.3390/md21110560/s1. Figure S1: Pearson correlations between selected induced lipids in the *m*PhoD/WT comparison; Table S1: Information of all lipid molecules detected in *P. tricornutum*; Table S2: Differential lipids detected in the *m*PhoD/WT comparison; Table S3: Differentially expressed genes (DEGs) involved in the lipid metabolism in the *m*PhoD/WT comparison; Table S4: Expression level of DEGs involved in fatty acid biosynthesis, arachidonic acid metabolism, LUFA synthesis, ceramide synthesis, and phosphatidylinositol signaling system in the *m*PhoD/WT comparison.

## Abbreviations

FA	Fatty acid
PUFA	Polyunsaturated fatty acid
LC-PUFA	Long-chain polyunsaturated fatty acid
LPC	Lysophosphatidylcholine
LPE	Lysophosphatidylethanolamine
LPI	Lysophosphatidylinositol
LPG	Lysophosphatidylglycerol
Cer	Ceramide
MGMG	Monogalactosylmonoacylglycerol
PIP2	Phosphatidylinositol bisphosphate
DHA	Docosahexaenoic acid
EPA	Eicosapentaenoic acid
ARA	Arachidonic acid
