# Supplementary material for: Alkaline Phosphatase PhoD Mutation Induces Fatty Acid and Long-Chain Polyunsaturated Fatty Acid (LC-PUFA)-Bound Phospholipid Production in the Model Diatom Phaeodactylum tricornutum
Source: Mar Drugs. 2023 Oct 26;21(11):560. doi: 10.3390/md21110560 (PMC10672530; doi:10.3390/md21110560)
Supplement: Supplementary file 1 [file marinedrugs-21-00560-s001.zip › Supplemental material.pdf]

Alkaline Phosphatase PhoD Mutation Induces Fatty Acid and Long-Chain Polyunsaturated Fatty Acid (LC-PUFA)-Bound Phospholipid Production in the Model Diatom *Phaeodactylum tricornutum*

Kaidian Zhang*†, Jiashun Li†, Jie Cheng, Senjie Lin*

The following Supporting Information is available for this article:

Fig. S1. Pearson correlations between selected induced lipids in the *m*PhoD/WT comparison.

Table S1. Information of all lipid molecules detected in *P. tricornutum*.

Table S2. Differential lipids detected in the *m*PhoD/WT comparison.

Table S3. Differentially expressed genes (DEGs) involved in the lipid metabolism in the *m*PhoD/WT comparison.

Table S4. Expression level of DEGs involved in fatty acid biosynthesis, arachidonic acid metabolism, LUFA synthesis, ceramide synthesis, and phosphatidylinositol signaling system in the *m*PhoD/WT comparison.

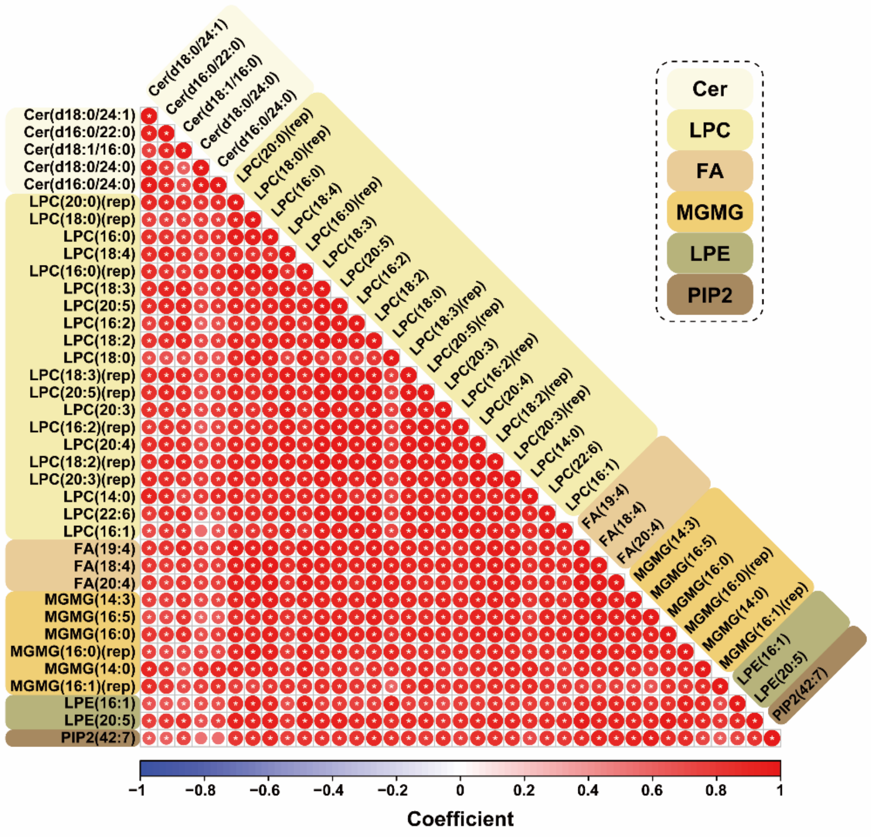

Fig. S1. Pearson correlations between selected induced lipids in the *m*PhoD/WT comparison. Red colour represents positive correlation. Asterisks in the middle of the circle represent $p < 0.05$.
